# Designer Biopolymers: Self-Assembling Proteins and Nucleic Acids

**DOI:** 10.3390/ijms21093276

**Published:** 2020-05-06

**Authors:** Ayae Sugawara-Narutaki, Yukiko Kamiya

**Affiliations:** 1 Department of Energy Engineering, Graduate School of Engineering, Nagoya University, Furo-cho, Chikusa-ku, Nagoya 464-8603, Japan; 2 Department of Biomolecular Engineering, Graduate School of Engineering, Nagoya University, Furo-cho, Chikusa-ku, Nagoya 464-8603, Japan; yukikok@chembio.nagoya-u.ac.jp

Nature has evolved sequence-controlled polymers such as DNA and proteins over its long history. The recent rapid progress of synthetic chemistry, DNA recombinant technology, and computational science, as well as the elucidation of molecular mechanisms in biological processes, drive us to design ingenious polymers that are inspired by naturally occurring polymers but surpass them in specialized functions. The term “designer biopolymers” refers to polymers consisting of biological building units such as nucleotides, amino acids, and monosaccharides in a sequence-controlled manner. They may contain non-canonical nucleotides/amino acids/monosaccharides, or they may be conjugated to synthetic polymers to acquire specific functions in vitro and in vivo.

This special issue, entitled “Designer Biopolymers: Self-Assembling Proteins and Nucleic Acids” particularly focuses on the self-assembling aspect of designer biopolymers. Self-assembly is one common feature in biopolymers used to realize their dynamic biological activities and is strictly controlled by the sequence of biopolymers. In a broad sense, the self-assembly of biopolymers includes a double-helix formation of DNA, protein folding, and higher-order protein assembly (e.g., viral capsids). Designer biopolymers are now going beyond what nature evolved: researchers have generated DNA origami, protein cages, peptide nanofibers, and gels. This special issue assembles three review papers and seven research articles on the latest interdisciplinary work on self-assembling designer biopolymers.

The review paper by Lee et al. covers design, self-assembly, and application of various designer peptides including dipeptides, amphiphilic peptides, and cyclic peptides [1]. These peptides are especially useful in drug delivery systems and tissue engineering. The in-cell self-assembly of peptides, termed “reverse engineering of peptide self-assembly,” is highlighted as a new approach to deliver peptide-based nanostructures to cells. The protein-based self-assembly system is reviewed by Nesterenko et al. [2]. The building block, ZT, is a complex from two titin Z1Z2 domains and telethonin. The Z1Z2 double tandem proteins (Z1Z2–Z1Z2) and telethonins co-assemble into polymeric nanostructures. They are robust scaffolds that can be genetically functionalized with full-length proteins and bioactive peptides prior to self-assembly. Functionalized ZT polymers successfully sustain the long-term culturing of stem cells. The review paper by Pereira et al. focuses on designer polymers based on cyanobacterial extracellular polymeric substances (EPS) [3]. The cyanobacterial EPS, mainly composed of heteropolysaccharides, emerges as a valid alternative to address several biotechnological and biomedical challenges. The review covers the characteristics and biological properties of cyanobacterial EPS, approaches to improving the production of the polymers by metabolic engineering, strategies for their extraction, purification, and genetic/chemical functionalization, and their use in scaffolds and coatings.

Two research articles address the important self-assembly phenomena of natural peptides. Antimicrobial peptides (AMPs) are a diverse group of membrane-active peptides that can interact with target membranes and can cause cell death by disturbing the membrane structure. Petkov et al. report molecular dynamics simulations studies on the solution behaviour of an AMP, bombinin H2 [4]. The simulation results show that bombinin H2 rapidly self-associate when multiple peptide chains are present in the solution, and the aggregation promotes further folding of bombinin H2 towards the biologically active shape. This study suggests that AMPs reach the target membrane in a functional folded state and are able to effectively exert their antimicrobial action. Amyloidogenic peptides including Aβ_1–40_, α-synuclein, and β2 microglobulin are regarded as hallmark peptides associated with key onset mechanisms of neurodegenerative diseases. Yokoyama et al. report pH-dependent adsorption of these peptides onto gold nanoparticles [5]. Nano-scale geometrical simulation with a simplified protein structure (i.e., prolate) represents peptide adsorption orientation on a gold colloid, indicating the presence of electrostatic intermolecular and gold-peptide interactions. 

Two other articles use engineered peptides to control inorganic mineralization or peptide-cell interactions. Kojima et al. describe the effects of peptide secondary structures on hydroxyapatite (HAp) biomineralization [6]. HAp-peptide composites containing a β-sheet forming peptide show a higher adsorption ability for basic proteins than those containing an α-helix forming peptide, most likely due to higher carboxy group density at the surfaces of former composites. Nanofibers formed from antigenic peptides conjugating to β-sheet-forming peptides have been recognized as promising candidates for next-generation nanoparticle-based vaccines. Waku et al. demonstrate that the hydrophilic-hydrophobic balance of peptide nanofibers affects their cellular uptake, cytotoxicity, and dendritic cell activation ability, which will provide useful design guidelines for the development of effective nanofiber-based vaccines [7]. 

In nature, proteins are often designed to form filamentous and circular oligomers to play their function. The articles from Sekiguchi et al. and Satoh et al. provide mechanistic insights into an assembly system of 20S proteasome, which is a huge protein complex consisting of homologous subunits α1–α7 and β1–β7 [8,9]. The correct assembly of proteasome subunits is essential for the function. Sekiguchi et al. comprehensively characterize the oligomeric states of the α1–α7 [8]. The results provide potential mechanisms on how the assembly and disassembly of proteasomal α subunits are controlled. Assembly of some subunits are assisted by chaperones. Satoh et al. have created a model of PAC3-PAC4 associated with α4–α5–α6 subcomplex based on their biophysical and biochemical analyses, providing functional mechanisms of the PAC3-PAC4 heterodimer as a molecular matchmaker underpinning the α4–α5–α6 subcomplex during α-ring formation [9]. Their findings open up new opportunities for the creation of artificial protein-assembling machine and also design of inhibitors of proteasome biogenesis.

Creation of artificial nucleic acids and applications are key trends. Mercurio et al. use a peptide nucleic acid (PNA), which is the neutral pseudo-peptide backbone, based on *N*-(2-aminoethyl) glycine units for the downregulation of miRNA function in the ascidian *Ciona intestinalis*. They have evaluated the expression level of miR-7 in a developing stage dependent manner and inhibitory effect of anti-miR-7, which will provide potential usage of PNA for basic research and therapeutics [10].

As shown by this special issue, self-assembly of biopolymers has a great impact on a variety of research fields including molecular biology, neurodegenerative diseases, drug delivery, gene therapy, regenerative medicine, and biomineralization. Designer biopolymers will help researchers to better understand biological processes as well as to create innovative molecular systems. We believe that this issue will provide readers with new ideas in their molecular design strategies for frontier research.

## References

[1] Lee S., Trinh T.H., Yoo M., Shin J., Lee H., Kim J., Hwang E., Lim Y.-B., Ryou C. (2019). Self-Assembling Peptides and Their Application in the Treatment of Diseases. Int. J. Mol. Sci..

[2] Nesterenko Y., Hill C.J., Fleming J.R., Murray P., Mayans O. (2019). The ZT Biopolymer: A Self-Assembling Protein Scaffold for Stem Cell Applications. Int. J. Mol. Sci..

[3] Pereira S.B., Sousa A., Santos M., Araújo M., Serôdio F., Granja P., Tamagnini P. (2019). Strategies to Obtain Designer Polymers Based on Cyanobacterial Extracellular Polymeric Substances (EPS). Int. J. Mol. Sci..

[4] Petkov P., Lilkova E., Ilieva N., Litov L. (2019). Self-Association of Antimicrobial Peptides: A Molecular Dynamics Simulation Study on Bombinin. Int. J. Mol. Sci..

[5] Yokoyama K., Brown K., Shevlin P., Jenkins J., D’Ambrosio E., Ralbovsky N., Battaglia J., Deshmukh I., Ichiki A. (2019). Examination of Adsorption Orientation of Amyloidogenic Peptides Over Nano-Gold Colloidal Particle Surfaces. Int. J. Mol. Sci..

[6] Kojima S., Nakamura H., Lee S., Nagata F., Kato K. (2019). Hydroxyapatite Formation on Self-Assembling Peptides with Differing Secondary Structures and Their Selective Adsorption for Proteins. Int. J. Mol. Sci..

[7] Waku T., Nishigaki S., Kitagawa Y., Koeda S., Kawabata K., Kunugi S., Kobori A., Tanaka N. (2019). Effect of the Hydrophilic-Hydrophobic Balance of Antigen-Loaded Peptide Nanofibers on Their Cellular Uptake, Cellular Toxicity, and Immune Stimulatory Properties. Int. J. Mol. Sci..

[8] Sekiguchi T., Satoh T., Kurimoto E., Song C., Kozai T., Watanabe H., Ishii K., Yagi H., Yanaka S., Uchiyama S. (2019). Mutational and Combinatorial Control of Self-Assembling and Disassembling of Human Proteasome α Subunits. Int. J. Mol. Sci..

[9] Satoh T., Yagi-Utsumi M., Okamoto K., Kurimoto E., Tanaka K., Kato K. (2019). Molecular and Structural Basis of the Proteasome α Subunit Assembly Mechanism Mediated by the Proteasome-Assembling Chaperone PAC3-PAC4 Heterodimer. Int. J. Mol. Sci..

[10] Mercurio S., Cauteruccio S., Manenti R., Candiani S., Scarì G., Licandro E., Pennati R. (2019). miR-7 Knockdown by Peptide Nucleic Acids in the Ascidian Ciona intestinalis. Int. J. Mol. Sci..

